# Legal Decision, Malpractice

**Published:** 1854-05

**Authors:** 


Legal Decision, Malpractice.—We copy the following important decision in a case of alleged *malpractice*, from the *Medical Reporter:*

The following opinions, delivered by Judges Woodward, Black, and Lewis, in reference to the subject of skill in a Physician or Surgeon, in attending a patient, to free him from the charge of malpractice, should he fail to effect a cure, will be read with interest. They are of more than ordinary importance, and it will be seen, overturns the law of the Court below.

Dr. Alexander G. McCandless *vs.* James McWha.—Error to Common Pleas of Beaver county.—Woodward, J.—This was an action on the case by the defendant in error, a respectable physician and surgeon, for malpractice in setting a broken leg of the plaintiff, and the only question of any importance, presented for our consideration, is whether the Court erred in charging "that the defendant was bound to bring to his aid the skill necessary for a surgeon, to set the leg so as to make it straight, and of equal length of the other when healed; and if he did not, he was accountable for damages, just as a stone mason of brick layer would be, in building a wall of poor materials and the wall fell down; or if they built a chimney, and it should smoke by reason of a want of skill in its construction."

It is impossible to sustain this proposition. It is not true in the abstract, and if it were, it was inapplicable to the circumstances of the case under investigation. The implied contract of a physician or surgeon is not to cure—to restore a fractured limb to its natural perfectness—but to treat the case with diligence and skill. The fracture may be so complicated that no skill vouchsafed to man, can restore original straightness and length, or the patient may, by wilful disregard of the surgeon's directions, impair the effect of the best conceived measures. He deals not with insensate matter, like the stone mason or brick layer, who can choose their materials and adjust them according to mathematical lines, but he has a suffering human being to treat, a nervous system to tranquilize, and a *will* to regulate and control. The evidence before us marks this strong distinction between surgery and masonry, and shows how the Judge's inapt illustration was calculated to lead away the jury from the true point of the cause.

Dr. Duncan describes the fracture as an oblique comminuted one, of the *tibia* and *fibula* of the leg, about half way between the ancle and the knee, and he says that on one occasion, when he was present at a dressing of the limb, he heard Dr. McCandless complain that McWha had loosened the bandages, and he told him that if he loosened them, his leg might be shortened, but McWha justified his act because his leg was painful. Now upon such a state of facts, the question was, not whether the Doctor had brought to the case skill enough to make the leg as straight and long as the other, but whether he had employed such reasonable skill and diligence as are ordinarily exercised in his profession. For less than this he is responsible in damages, but if he be held to the measure laid down by the Court below, the implied contract amounts, on his part, to a *warranty of cure*, for which there is no authority in law. In a fracture like this, a shortening of the limb is sometimes an inevitable consequence. Dr. Dorsey, in his Elements of Surgery, speaking of broken legs below the knee, says:—"The fracture of both bones is most frequent—it may be transverse or oblique, simple or compound, comminuted or single. The fragments are occasionally displaced in every direction. In transverse fractures there is generally no shortening of the limb, but in those which are oblique, the leg is generally shortened." And from Ferguson's System of Practical Surgery, cited in the argument, we learn that the fracture in the tibia may be oblique, and the fragments, two or more, may have a constant tendency to become displaced—there may be great irritability of the muscles, particularly during the early part of the treatment—great restlessness of the patient, or unwillingness to submit to the requisite confinement; in short, a vast variety of circumstances likely to cause difficulty in the treatment. Not to multiply authorities, these are sufficient to show that the rule prescribed by the Court is too rigid for this class of cases—that shortening of the leg may result from the most careful and approved practice, or from the misconduct of the patient. Nothing can be more clear than that it is the duty of the patient to co-operate with his professional advisers, and to conform to the necessary prescriptions; but if he will not, or under the pressure of pain cannot, his neglect is his own wrong or misfortune, for which he has no right to hold his surgeon responsible. No man may take advantage of his own wrong, or charge his misfortunes to the account of another.

We do not mean to intimate an opinion that this case was properly treated, or that the leg could not have been restored to the length of its fellow; but in view of the diversified circumstances that attend cases of this sort, it was very important that the true rule of professional responsibility should have been given to the jury, with instructions that they should inquire, from all the facts in proof, whether the defendant had come up to it, or stopped short of it.

We have stated the rule to be reasonable skill and diligence, by which we mean such as thoroughly educated surgeons ordinarily employ. If more than this is expected, it must be expressly stipulated for, but this much every patient has a right to demand in virtue of the implied contract which results from entrusting his case to a person holding himself out to the world as qualified to practice this important profession. If a patient applies to a man of *different occupation* or employment, for his assistance who either does not exert his skill, or administers improper remedies to the best of his ability, such person is not liable in damages; but if he applies to a *Surgeon*, and he treats him improperly, he is liable to an action, even though he undertook gratis to attend the patient, because his situation implies skill in surgery. Per. Heath J., in Shiels v. Blackburn, 1 Hen. Blac.; 161 Seare v. Prentice, 8 East., 348.

The principle is contained in the pithy saying of Fitzherbert, that it is the duty of every artificer to exercise his art rightly and truly, as he ought. This is peculiarly the duty of professional practitioners, to whom the highest interests of man are often necessarily entrusted. The law has no allowance for quackery. It demands *qualification* in the profession practiced—not extraordinary skill, such as belongs only to a few men of rare genius and endowments, but that degree which ordinarily characterizes the profession. And in judging of this degree of skill, in a given case, regard is to be had to the advanced state of the profession at the time. Discoveries in the natural sciences for the last half century, have exerted a sensible influence on all of the learned professions, but especially on that of medicine, whose circle of truths has been relatively much enlarged. And, beside, there has been a positive progress in that profession, resulting from the studies, the experiments, and the diversified practices of its professors. The patient is entitled to the benefit of these increased lights. The physician or surgeon who assumes to exercise the healing art, is bound to be up to the improvements of the day. The standard of ordinary skill is on the advance, and he who would not be found wanting, must apply himself with all diligence to the most accredited sources of knowledge.

If, in view of the principles here stated, Dr. McCandless shall be found, on re-trial, to have performed his whole duty to his patient, and that any defects in the limb are due to the patient's fault, or to the peculiarities of the fracture there ought to be no recovery in damages. But if the blemish be fairly attributable to professional negligence, the jury should assess the damages.

The only remaining error assigned is scarcely worthy of notice. The action depended so entirely on its own circumstances that the observation of the Court as to the policy of such suits was irrelevant, and we may fairly presume harmless. But for misdirection on the other point the judgment is reversed, and a *venire de novo awarded.*

McCandless *vs.* McWha.—Black.—We all concur in the law of this case. The Judge, in his charge, fell into an error in stating the amount of skill required in the treatment of this case. We reverse for that reason. But when we decide the legal point we are done with it. We are not authority on questions of surgery. Our hands are abundantly full of questions which belong to our own profession, without volunteering opinions on sciences which relate to others. I think it necessary to say this in order to prevent the Court below, on second trial, from supposing that we intend to give them any instructions on matters in which we have no jurisdiction.

McCandless *vs.* McWha.—Lewis J.—Without dissenting from the able opinion of Mr. Justice Woodward, I make the following additional remarks: The case is peculiar, and relates to matters of such general interest as to justify this course. The Court below charged the jury that "the defendant was bound to bring to his aid the skill necessary for a surgeon to set the leg so as to make it straight and of equal length with the other when healed; and if he did not he was accountable in damages, just as a stone mason or bricklayer would be in building a wall of poor materials and the wall fell down; or if they built a chimney, and it would smoke by reason of a want of skill in its construction." This is the error complained of, and it seems to be thought that the Court, in giving this instruction, held the surgeon bound, under all circumstances, to cure the fractured leg so as to "make it straight and of equal length with the other when healed." I do not so understand the language of the Judge. He only held the surgeon bound to "*bring to his aid*" the skill necessary for the purpose. If the fracture in question was one which might have been restored by the exercise of ordinary skill, there was no error in requiring its exercise from one who held himself out as possessing it, and received compensation for his services in consequence of his represented professional ability. This brings us to the question: Was the injury one which might have been cured by the exercise of ordinary surgical skill? To decide this question we must have a description of the fracture. The evidence given has not been brought up by the bill of exceptions, and the defendant in error objects to that part of it which has been inserted in the paper-book without being certified as correct. The only testimony presented for consideration here, by the plaintiff in error, is the deposition of Dr. Duncan, who was his student at the time of the injury, and visited the patient in company with his preceptor after the first visit of the latter. This witness describes the injury to be "an oblique comminuted fracture of the *tibia* and *fibula*, nearly half way from the ancle to the knee, or thereabouts;" and informs us, in speaking of the treatment of it by Dr. McCandless, that "there were splints on the fore and back parts of the leg, reaching from the ankle to the knee, to keep up extension and counter-extension." Dr. McCandless, on this visit, complained that the patient had "disturbed the bandages and dressing *by loosening them;*" and the patient "defended the act *of loosening the bandages*, because the leg was painful." The witness further states that the leg, at this time, was "considerably swollen."

We have no precise account of the manner in which the splints were secured, so as to "keep up the extension and counter-extension," for which the witness tells us they were designed. I am unable to comprehend how splints "reaching only from the knee to the ankle," could be applied to such a purpose without manifest danger of injury by means of the attachments which would be necessary to produce the result. *Extension*, as used among surgeons, is the force exerted on the lower fragment, in order to bring its superior extremity lower than the inferior extremity of the superior fractured portion; and *counter-extension* is a resisting force which prevents the whole limb, or even the body, from obeying the force of extension. The attachment, by means of a circular bandage at the ankle, for the purpose of extension, and that at the knee, for the purpose of counter-extension, would tend to impede the circulation, particularly the venous return, (which ought not to be obstructed,) and would irritate the parts so as to produce great pain and probable injury. Professor Boyer, in his Lectures on Diseases of the Bones, recommends that the splints should be long enough to extend from the knee to *a short distance beyond the sole of the foot*, and that they should rest perpendicularly on their edges, and a third splint on the anterior portion of the leg. Professor Miller, in his Principles of Surgery, states that the splints should "*invariably* be of sufficient length to *command the neighboring joints*, otherwise, by rotation, re-displacement will certainly take place." Dr. Hutchinson recommends splints extending from the knee *six or eight inches below the sole of the foot*, so as to dispense with irritating attachments at the ankle. But Professor Dorsey, whose skill and experience entitles his opinion to great respect, in his work on Surgery, informs us that even Hutchinson's convenient method is found to produce great irritation, and to cause the leg to swell from the pressure of the circular bandages; and that when this happens, in oblique fractures of the leg, (such as the case in question,) *the long splint of*

*Desault must be substituted*, and the *counter-extension made at the pelvis*, in the same manner as in the case of a fractured thigh except that the leg must be dressed with the bandage of strips." In fractures of the thigh, permanent extension is usually effected by means of a long splint, acted on by a band attached to its upper extremity, and passed over the perineum, by the tightening of which the splint and the limb are pushed steadily downwards. By the addition of a shorter splint, but long enough nevertheless to extend from the perineum to six or eight inches beyond the sole of the foot, united at the lower extremity to the long splint by means of a cross piece, the extending force could be applied to the ankle by attachments to the cross-piece, in such a manner as to avoid irritation or other injury. But according to the opinion of eminent surgeons, "a short splint, extending a little above and below the fracture only, is not only an absurdity but a mischievous absurdity."—Miller's Prin. Surgery, 507.

Entertaining these views of the case, I am bound to say that the plaintiff in error has failed to satisfy me, either upon philosophical principles, or by surgical authority, that the means made use of, for the purpose of producing "extension and counter-extension," were adequate or even proper for the purpose. If this was a case in which such extension by artificial means was not required, the mere want of adaptation of the means to that end would be immaterial. But we must remember that the fracture was oblique, not transverse—that it was comminuted—that is, the bones were broken not only at one point, but many—and that both the *tibia* and *fibula* were thus fractured. Under these circumstances, in preventing the shortening of the limb by the contraction of the muscles, no reliance could be placed upon the bones thus broken into fragments. The necessity of supplying the place of these natural splints, by artificial means, must therefore have been manifest to a surgeon of ordinary skill in his profession. But in addition to the application of means not sufficient to produce the result which was indispensable to a proper restoration of the leg, there is reason to believe, judging solely from the imperfect view of the evidence presented by the plaintiff in error himself, that the short splints were applied by attachments above and below the fracture, so as to impede the circulation, to irritate the parts, to cause the limb to be "considerably swollen," and to produce so much pain that the patient, notwithstanding the strong motive which he had to submit to any treatment likely to effect a perfect recovery, "loosened the bandages because the leg was painful." If this was the case, whatever may be thought of the propriety of the original application of these means of extension, their continuance and the neglect to adopt others less liable to objection, was *prima facia* evidence of a want of surgical skill, and if not explained to the satisfaction of the jury, the defendant below ought to answer in damages for the injury.

A patient is bound to submit to such treatment as his surgeon prescribes, provided the treatment be such as a surgeon of ordinary skill would adopt or sanction. But if it be painful, injurious and unskillful, he is not bound to peril his health, and perhaps his life, by submission to it. It follows, that before the surgeon can shift the responsibility from himself to the patient, on the ground that the latter did not submit to the course recommended, it must be shown that the prescriptions were proper, and adapted to the end in view. It is incumbent on the surgeon to satisfy the jury on this point, and, in doing so, he has the right to call to his aid the science and experience of his professional brethren. It will not do to cover his own want of skill by raising a mist out of the refractory disposition of the patient.

The intemperate habits of the patient are also relied upon here. But this furnishes no excuse for the want of skill in the surgeon. On the contrary, it was a circumstance calculated to admonish him that the case called for more skill and care than cases of less difficulty demand. We are, therefore, brought back to the main questions in the cause.

1. Did the surgeon exercise ordinary skill and care in his treatment of the patient? If he did, he is not liable. If he did not, he is.

2. Was the injury one, which, under all the circumstances, might have been perfectly cured by ordinary surgical skill and care? If it was, and the surgeon failed in his duty in this respect, the damages ought, at least, to be commensurate with the injury. If the injured leg was not susceptible of a more perfect restoration, the surgeon would nevertheless be liable for any unnecessary pain or delay occasioned by the application of unskillful and improper remedies.

Although the error assigned may not be fully sustained, we have nevertheless a right, in our discretion, to reverse for an error not assigned, if it is believed to involve an important principle, or to affect the justice of the case.

In the charge the Court told the jury in substance, that the surgeon was bound to bring to his aid the skill necessary to effect a perfect restoration of the leg. The propriety of this instruction depended upon the question whether the injury was one which under all circumstances, a surgeon of ordinary skill might have perfectly cured. This was a question of *fact*, which should have been submitted to the jury. Plain as the question may seem, it is not a matter of *law*, the decision of which can be taken from them and assumed by the Court. There was, therefore, error in giving the peremptory and unqualified direction which withdrew this part of the case from the jury. But there are errors of omission as well as those of commission. When the Judge spoke of the obligations of the surgeon to bring to his aid the necessary skill, he ought to have enforced the co-relative duties of the patient to submit to all the skillful and proper requirements of his professional attendant. When the jury were told, in effect, that the defendant was liable if he failed to exercise the skill necessary to a perfect restoration of the leg, they ought also to have been informed that if he exercised ordinary skill and care, he is not responsible for the disastrous result which ensued. Where a case turns upon a question of fact, the jury should be advised of the conclusions of law which apply to each aspect of it. The object of investigations is to enable the jury to form an enlightened judgment on the whole case. The errors of commission and omission referred to, tended to give the jury a one-sided view of the controversy, and when considered in connexion with the facts that a *professional man* was on trial before a jury of laymen, and that the Court, instead of guarding him, as in duty bound, against the prejudices likely to arise in such cases, actually indulged in a strain of remark calculated to inflame them, it is our duty to correct all the errors within our reach. The remarks complained of in the second assignment of error, affirm no principle of *law*, and are therefore not the subject of review here further, than as they suggest the propriety of exercising a prudent discretion in regard to matters which are subject to review.

It is important to the interests of society, that the profession entrusted with the preservation of the health and lives of the community should be held to a strict rule of accountability. Men of true science will not object to this. They court investigation. But the incompetent practitioner and the designing empiric, "love darkness rather than light," and the sooner they are driven by judicial scrutiny, into other pursuits for which they are better qualified, and where they can do less mischief, the better for the public welfare. But it is equally important that professional services should be fairly treated, and that true skill and worth should receive the firm protection of the law. All men have a right to the instructions which make in their favor. But the exigency of the surgeon's case rendered them indispensable on the present occasion. The difficulties which seem to stand in his way are sufficient, without aggravating them by withholding the proper instructions in his favor.

For these reasons I am in favor of reversing the judgment and awarding a *venire de novo.*

